# A Pharmaco-Technical Investigation of Thymoquinone and Peat-Sourced Fulvic Acid Nanoemulgel: A Combination Therapy

**DOI:** 10.3390/gels8110733

**Published:** 2022-11-10

**Authors:** Rahmuddin Khan, Mohd Aamir Mirza, Mohd Aqil, Nazia Hassan, Foziyah Zakir, Mohammad Javed Ansari, Zeenat Iqbal

**Affiliations:** 1 Department of Pharmaceutics, School of Pharmaceutical Education & Research (SPER), Jamia Hamdard, New Delhi 110062, India; 2 Department of B. Pharm (Ayurveda), School of Pharmaceutical Sciences, Delhi Pharmaceutical Sciences and Research University, New Delhi 110017, India; 3 Department of Pharmaceutics, College of Pharmacy, Prince Sattam Bin Abdulaziz University, Al-Kharj 16278, Saudi Arabia

**Keywords:** nanoemulsion, pseudo-ternary phase diagrams, enhanced stability, thymoquinone, ultrasonication, Box–Behnken design

## Abstract

Thymoquinone has a multitude of pharmacological effects and has been researched for a wide variety of indications, but with limited clinical success. It is associated with pharmaco-technical caveats such as hydrophobicity, high degradation, and a low oral bioavailability. A prudent approach warrants its usage through an alternative dermal route in combination with functional excipients to harness its potential for treating dermal afflictions, such as psoriasis. Henceforth, the present study explores a nanoformulation approach for designing a fulvic acid (peat-sourced)-based thymoquinone nanoemulsion gel (FTQ-NEG) for an enhanced solubility and improved absorption. The excipients, surfactant/co-surfactant, and oil selected for the *o*/*w* nanoemulsion (FTQ-NE) are Tween 80/Transcutol-P and kalonji oil. The formulation methodology includes high-energy ultrasonication complemented with a three-dimensional/factorial Box–Behnken design for guided optimization. The surface morphology assessment through scanning/transmission electron microscopy and fluorescence microscopy revealed a 100 nm spherical, globule-like structure of the prepared nanoemulsion. Furthermore, the optimized FTQ-NE had a zeta potential of −2.83 ± 0.14 Mv, refractive index of 1.415 ± 0.036, viscosity of 138.5 ± 3.08 mp, and pH of 5.8 ± 0.16, respectively. The optimized FTQ-NE was then formulated as a gel using Carbopol 971^®^ (1%). The in vitro release analysis of the optimized FTQ-NEG showed a diffusion-dominant drug release (Higuchi model) for 48 h. The drug permeation flux observed for FTQ-NEG (3.64 μg/cm^2^/h) was much higher compared to that of the pure drug (1.77 mg/cm^2^/h). The results were further confirmed by confocal microscopy studies, which proved the improved penetration of thymoquinone through mice skin. Long-term stability studies of the purported formulation were also conducted and yielded satisfactory results.

## 1. Introduction

The evolving monopoly of herbal/traditional medicines in the form of phytoconstituents, bioactive molecules, and extracts for healthcare has attracted both researchers and medical practitioners alike. Their multifarious attributes, such as the nutrient composition and antioxidant, anti-inflammatory, and therapeutic properties, have benefited around 80% of the world population [1]. Thymoquinone (TQ), a lipophilic benzoquinone, is one such phytoconstituent found in *Nigella sativa* (black cumin or black seed) of the family *Ranunculaceae*, which exhibits biological functions [1,2] such as the regulation of cell proliferation and inflammation, as well as antioxidant and other therapeutic activities [3,4,5]. The translational index of TQ from traditional medicines to clinical trials further complements its efficacy and potential against various ailments and conditions, such as psoriasis, cancer, inflammatory/auto-immune disorders, metabolic syndrome, and cytotoxicity. Despite its appreciable pharmacological potential, TQ has limited clinical success owing to its pharmaco-technical caveats, such as its hydrophobicity, thermal/photodegradation, low bioavailability, and sensitivity (pH, temperature, light), as these attributes significantly affect its formulation performance [6]. Thus, to advance the therapeutic benefits of TQ, a suitable formulation development is necessary in order to not only surpass physicochemical constraints but also complement the efficacy of TQ [7,8]. The intervention of nanotechnology seems promising, as it can enhance the API (active pharmaceutical ingredient) solubility, bioavailability, and kinetic stability. The advent and successful use of functional excipients are other pharmaceutical strategies used to enhance the spectrum of use of therapeutically promising entities such as TQ. Henceforth, the present work explores a fulvic acid-based thymoquinone nanoemulsion (FTQ-NE) for improved drug delivery, absorption/permeation, and retention. Fulvic acid (FA) is a naturally occurring chemical found in Earth’s soils, rock deposits, and bodies of water [9]. FA has been used for the delivery of medications for various reasons, such as its water solubility in all pH ranges, low molecular weight, and high oxygen and low carbon content [10]. Morphologically, FA is a pale yellow to yellowish-brown powder with a well-known capacity to convert minerals into a highly accessible state [11]. These tiny fulvic molecules are tasked with the critical role of dissolving and delivering essential minerals from the soil to the plants [12]. FA can act as an electron donor or receptor in a concentration-dependent manner and scavenge a range of free radicals. For example_,_ oxygen can be scavenged from oxygenated free radicals in vitro or incorporated into the structure of FA to stop the chain reaction [13]. The exploration of FA as a broad-spectrum antimicrobial has been gaining attention, and various research reports have demonstrated its potential against the biofilms of *Streptococcus mutans* (periodontal infection), multi-drug-resistant bacteria, and *Candida albicans* [14,15]. Therefore, the ability of FA to enhance the solubility of APIs is explored in the present work. FA forms complexes with APIs, which are marred by their low solubility/bioavailability, thus setting new research goals and a hypothetical basis, especially for the present work [16].

The purported formulation for the current work is nanoemulsions (NE), which are transparent, kinetically stable, smaller globules ranging from 20–200 nm [17]. Their nano-globular size and the presence of surfactants enable the emulsification of insoluble drugs to enhance dermal penetration and drug retention, consequently fortifying the topical drug delivery [18,19]. In contrast to other nano-based carrier dosage forms, NE offers multiple positive attributes, such as the increased solubility of hydrophilic/lipophilic drugs, simple fabrication, and longer shelf-life, and it aids in the administration of the topical delivery [20,21]. However, NE has a lower rate of drug retention on the skin during topical delivery due to its low viscosity. Thus, the incorporation of a gelling material is advisable. For the present work, Carbopol 971^®^ was added to the prepared FTQ-NE to develop a nanoemulgel with a high skin absorption, permeation, and retention [22]. Henceforth, the advantages of NE gel can be exploited to fulfil the present research requirements, capturing the goodness of TQ in a technologically defined NE system, akin to putting “new wine in an old bottle”. This is a highly plausible mechanism that can be used to exploit the existing therapeutic moieties and revisit the formulation design strategies in order to scientifically and systematically create newer formulations, without placing a great burden on the already-drying drug development pipeline. It is worth mentioning that NE is widely accepted due to its ease of production and adaptability [23]. Additionally, it has the highest drug encapsulation efficiency compared to its competitors and the potential to improve solubility and bioavailability [24].

## 2. Results and Discussions

### 2.1. Screening of Excipients

**Figure 1:**
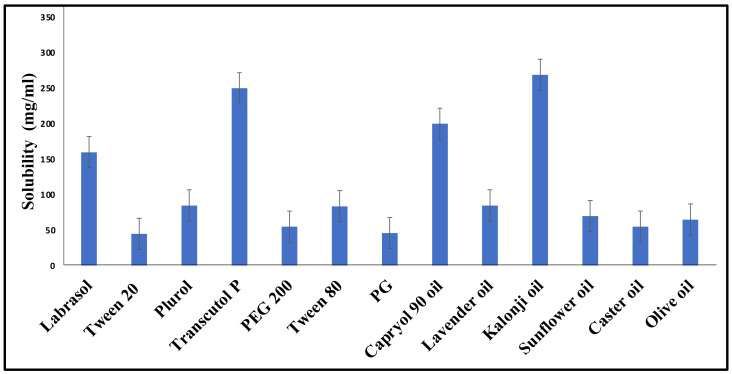
TQ solubility in various surfactants, co-surfactants, and oils.

The solubility of TQ in various excipients was investigated, and the findings are presented in Figure 1. Amongst the different experimental oils, the highest solubility (268.18 ± 0.20 mg/mL) of TQ was observed in the kalonji oil [25], making it a suitable oil phase for the purported formulation. Tween 80 and Transcutol-P were selected as the surfactant and co-surfactant, respectively, as they showed the maximum drug solubility, i.e., 83.23 ± 02.57 mg/mL and 250.32 ± 0.64 mg, respectively, as well as miscibility (Table 1). Furthermore, the combination (Smix ratio) of Tween 80 and Transcutol-P was selected via employing pseudo-ternary phase diagrams to produce a clear NE using the placebo formulations. Drug solubilization studies are critical for NE development, as they play a crucial role in the formulation stability, delivery, and maintenance. The selection of suitable excipients is also necessary to produce a transparent, clear, homogenous formulation with a high binding capacity for API (amongst other reasons) [26].

### 2.2. Formulation Development

#### 2.2.1. Preparation of a Placebo Nanoemulsion and Excipients Optimization

**Figure 2:**
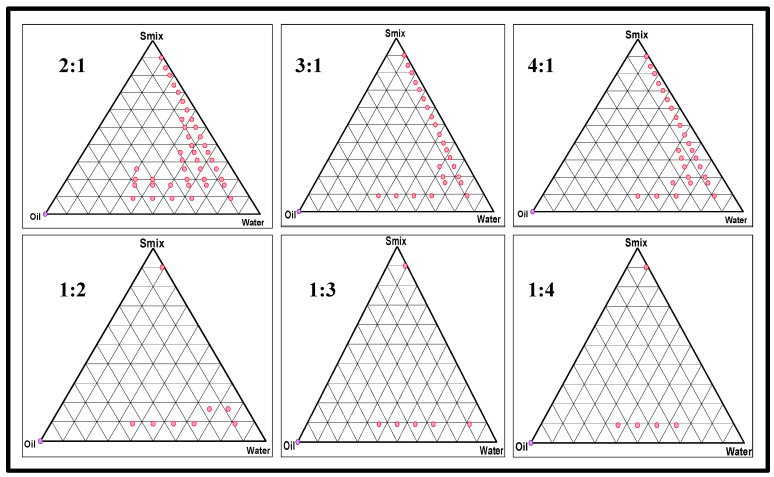
Pseudo-ternary phase diagrams for various Smix ratios.

The combination of the selected surfactants was further evaluated for the Smix ratio using a pseudo-ternary phase diagram (Figure 2), in which a dotted color represents an NE region. As observed, the Smix ratio of 1:4 produced the lowest nanoemulsion zone, whereas a Smix ratio of 2:1 produced the highest nanoemulsion zone. This could be due to a high HLB of Tween-80 and the fact that, subsequently, when the Tween 80 concentration is increased, the HLB of the Smix also increases. However, this explanation does not comply with a Smix ratio of 3:1, in which an increase in the Tween 80 concentration enhances the viscosity simultaneously, leading to droplet disruption and breakage in the NE [27]. The selected Smix ratio was then used to produce various placebo formulations, as the pseudo-ternary phase diagrams were also helpful in identifying the minimum and maximum proportions of oil and Smix to be used to obtain a clear, transparent, and homogenous NE. Notably, of the many combinations tested for an *o*/*w* NE preparation, only two were taken for further analysis, in which the minimum ranges of the oil phase and Smix were 1–2.5% and 16–18%, respectively. Below a concentration of 1%, the oil phase was incapable of solubilizing the required amount of API. At a concentration higher than 2.5%, it exceeded the percentage of water, leading to a formation of *w*/*o* NE. Additionally, a higher percentage of the oil phase requires a much higher concentration of Smix, i.e., more than 20%, leading to microemulsion rather than a purported NE [28].

#### 2.2.2. Preparation of a Drug-Loaded FTQ-NE

After carefully selecting various excipients and their optimized concentrations, a methodology suitable for the preparation of an FTQ-NE was adopted. The ultrasonication technique is a highly popular process employed for NE fabrication. It has the upper hand over the high-pressure homogenization technique due to the promised integrity of the NE droplets in terms of their shape and dispersion. In an HPH (high-pressure homogenizer), the size of a droplet may be smaller, but the surface can be irregular due to the high processing [29]. Additionally, the NE formed may have a high PDI, leading to polydisperse formulation [30]. Furthermore, an increase in temperature during the processing may adversely impact the formulation outcomes. Additionally, the high cost and maintenance may limit its commercial potential [31]. Henceforth, ultrasonication was selected as the method of choice for the fabrication of the FTQ-NE.

### 2.3. BBD: Mathematical Model Fitting and Optimization of FTQ-NE

**Figure 3:**
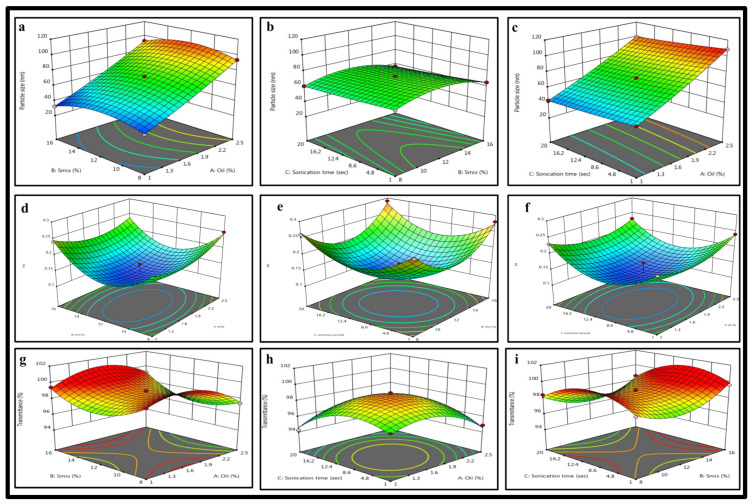
3D response surface depicting the interaction effect of the independent variables like oil, smix, sonication time on (**a**–**c**) particle size, (**d**–**f**) PDI, and(**g**–**i**) %transmittance.

For the present formulation, the independent variables and dependent responses selected were the oil concentration (%), Smix ratio (%), sonication time (s) and particle size (nm), polydispersity index, and transmittance (%), respectively. The ternary phase diagrams were utilized to select the high, medium, and low levels of the oil and surfactant concentrations required for DoE. The optimization design followed was the BBD, a response surface methodology approach used to predict the effects of independent variables on dependent responses (Table 2). The varied levels or experimental ranges for the selected independent variables are presented in (Table 3). As observed, all three dependent responses followed a polynomial quadratic model, with a non-significant lack of fit (*p* > 0.05) (Table 4). The observations of the BBD were recorded as 3D response surface graphs (Figure 3) for a comparative illustration of the interaction of each dependent response with two different independent variables [32]. Following are the detailed descriptions of each response:

#### 2.3.1. Effects of Independent Variables on the Particle Size

Its nano nature is one of the distinct features of FTQ-NE. The smaller the size is, the larger the surface area which ultimately affects the drug delivery, permeation, and bioavailability will be. However, the particle size might vary due to the effects of variables, as discussed further below. For example, with an increase in the oil concentration, the particle size increases due to the increased chain length (Figure 3), and as the present formulation is *o*/*w* NE, a larger oil droplet size may contribute to a large particle size as well [33]. It was also observed that the Smix ratio and sonication time do not affect the particle size (Figure 3a–c). As presented in Table 2, all 17 formulations had a particle size range of 32.26 ± 5.21–108.53 ± 7.14 nm.

#### 2.3.2. Effects of Independent Variables on the PDI

A PDI represents the formulation consistency in terms of the particle dispersion, i.e., monodisperse or polydisperse. Such a dispersion of the formulation particles might indicate their uniformity and degree of aggregation and may affect the drug distribution [34]. As shown in the BBD graphs, all three independent variables had considerable effects on the PDI (Figure 3d–f). Furthermore, all 17 formulations had PDI values in the range of 0.12–0.29 (Table 2), which was sufficiently suitable, as a high PDI of more than 0.3 generally signifies particle agglomeration, which may increase the particle size [35].

#### 2.3.3. Effects of Independent Variables on the %Transmittance

The %transmittance value reflects the amount of incident light that passes through a formulation or sample without being changed, i.e., it is neither absorbed nor scattered. Notably, the %transmittance values for clear solutions/dispersions are higher. In contrast, those for cloudier/turbid solutions/dense dispersions are lower, as more incident light can be transmitted through a clear sample rather than a turbid one. For an NE, the more refined/fine the particles are, the greater the transmittance will be. For the present formulation, each independent variable had a distinct impact on the %transmittance (Figure 3g–i), and the predicted 17 formulations elicited values ranging from 94.24 to 99.51%. Conclusively, the obtained range is suitable (transparency and clarity) and complies with the other dependent responses as well [36].

After evaluating each variable, the formulation, with a particle size of 72.34 ± 2.43 nm, PDI of 0.126 ± 0.014, and %transmittance of 98.99 ± 0.22%, was selected as the optimum formulation.

### 2.4. Thermodynamic Stability

Thermal stability studies, such as heating–cooling, centrifugation, and the freeze–thaw cycle, revealed that certain NE formulations were turbid, and some had phase separation (Table 5). Such instability might occur due to Ostwald ripening, a free-surface energy process in which small droplets combine via diffusion to form aggregates or larger droplets [37]. The formulations which did not show any thermodynamic instability were subjected to further physical characterization, as highlighted in Table 6. The collated results of both the thermodynamic and physical characterizations revealed the formulation F-A2 as the most suitable and optimized FTQ-NE.

### 2.5. Characterization of the Optimized FTQ-NE

#### 2.5.1. Dilution Test

**Figure 4:**
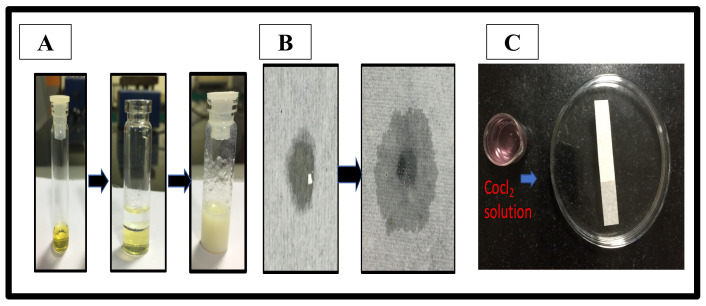
(**A**) Dilution test, (**B**) filter paper test, and (**C**) cobalt chloride test.

In the dilution test, the addition of oil leads to either cracking or phase separation. Thus, the optimized formulation, FTQ-NE (P-2), is of an *o*/*w* type (Figure 4A). Following this, the filter paper and cobalt chloride test was performed, which also revealed a similar NE type (*o*/*w*), depicting rapid spreading (Figure 4B) and change from blue to pink color on the filter paper (Figure 4C), respectively. Furthermore, the %transmittance of the optimized FTQ-NE was found to be 98.99% ± 0.42, confirming its transparency as well as nano-globule size. Furthermore, the obtained refractive index (RI) of 1.415 ± 0.036 of the optimized FTQ-NE also highlighted a less dense or clear isotropic formulation [38]. Lastly, the viscosity and pH of the optimized FTQ-NE were 138.5 ± 3.08 mp and 5.8 ± 0.16 mp, respectively.

#### 2.5.2. Particle Size, Polydispersity Index (PDI), and Zeta Potential

**Figure 5:**
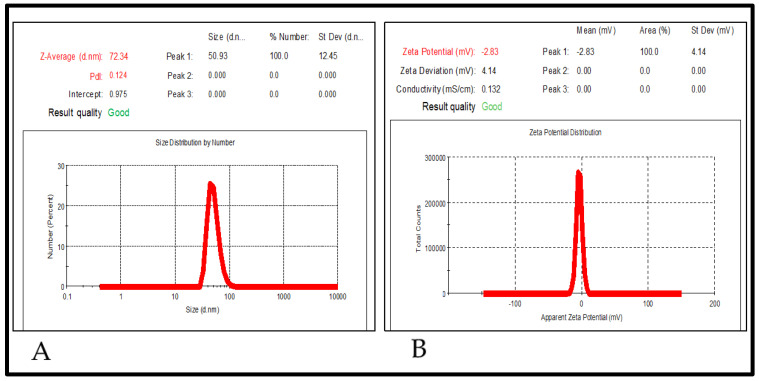
Particle size, polydispersity index (**A**) (PDI), and zeta potential (**B**) of FTQ-NE.

The optimized FTQ-NE revealed a droplet/particle size of 72.34 ± 2.43 nm (diameter), PDI of 0.126 ± 0.014 (Figure 5A), and zeta potential of −2.83 ± 0.14 mV (Figure 5B). As noted earlier, the nano size ensures an adequate surface area and more efficacious drug delivery, absorption, and retention. Generally, the NE falls in a diameter range of 5–200 nm, and based on the obtained results of the present FTQ-NE, the size range was concordant. A low PDI point to the monodisperse nature of a prepared formulation. Additionally, it indicates a high concentration of the surfactant, which forms a densely packed film at the oil–water interface, thus providing a greater stabilization/emulsification of the oil phase, rather than the co-surfactant. The negative value of the zeta potential could be due to the incorporation of non-ionic surfactants, which are expected to stabilize and lower the charge magnitude of the dispersed oil droplets [39].

#### 2.5.3. Differential Scanning Calorimetry (DSC)

**Figure 6:**
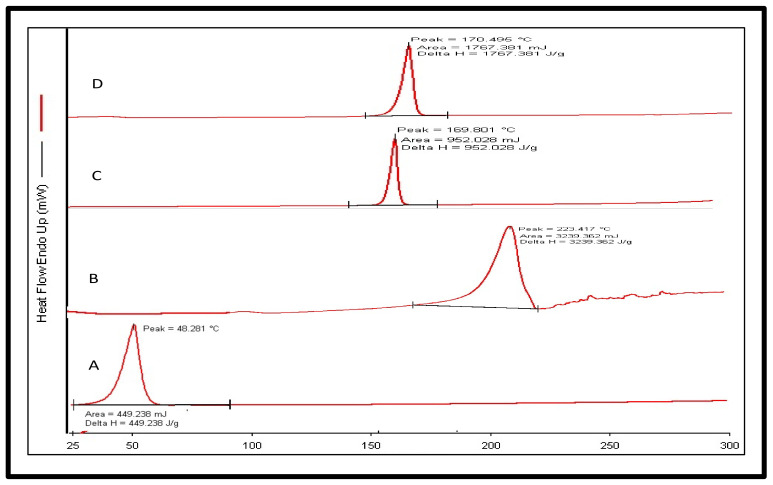
Overlay of differential scanning calorimetry (DSC) (**A**) (thymoquinone), (**B**) (fulvic acid), (**C**) (mannitol), and (**D**) (FTQ-NE).

The melting point of the pure drug was estimated to be 48.281 °C (area 449.238 mJ) (Figure 6), which corresponds to the reported melting range of 47 to 51 °C. The obtained peak was found to be endothermic, and the nature of the drug was crystalline. The DSC analysis of the FA reported a peak at 223.154 °C (area 3239.362 mJ) and a peak of the pure mannitol at 169.801 °C (area 952.028 mJ). However, in the DSC thermogram of the lyophilized FTQ-NE (optimized) formulation, only a single peak at 170.495 °C was noted (area 1767.381 mJ). The obtained peak was closest to that of the mannitol, a cryoprotectant which was added before the lyophilization of the optimized FTQ-NE. The single peak could be due to the high quantity of mannitol, while also depicting the transition of both the TQ and FA from a crystalline to amorphous state, which did not exhibit any significant peak. This could further accrue stability in terms of the TQ/FA leaching or precipitation within the proposed formulation [40].

#### 2.5.4. Fourier Transforms Infrared Spectroscopy (FTIR)

**Figure 7:**
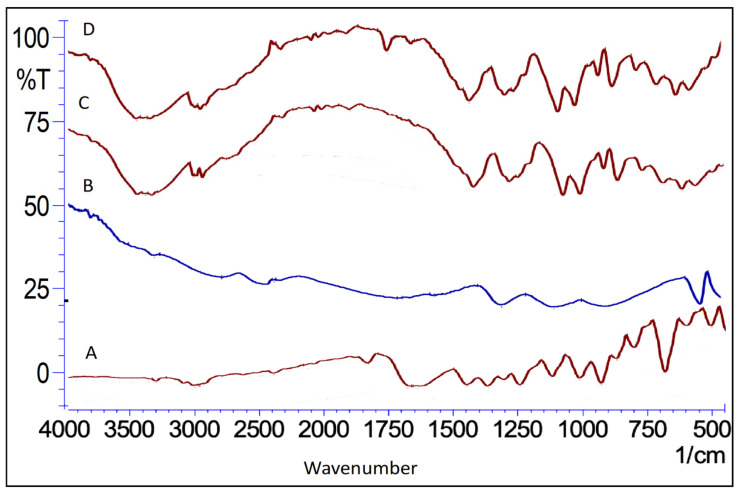
Overlay spectra showing the FTIR of (**A**) TQ, (**B**) FA, (**C**) mannitol, and (**D**) FTQ-NE.

The FTIR (KBr) spectra of the pure drug (Figure 7) showed characteristic peaks (cm^−1^) noted at 3253.91 (C-H stretching, alkene), 2927.94 (C-H stretching, methyl), 1653.00 (-C=O), 1446.61 (C-H bending, methyl), 935.48 (C-H wagging, alkene), and 877.61 (C=C, bending), respectively (Figure 7). Similarly, the spectra of the mannitol showed peaks (KBr) at 3399.68 (-OH), 3344.71 (-OH), 3288.77 (-OH), 3240.55 (-OH), 2975.33 (C-H, stretching), 2903.96 (C-H, stretching), 1423.53 (C-H, scissoring), and 1287.54 cm^−1^ (C-H, rocking). On the other hand, the absorption bands at 3650–3600 cm^−1^ (H-bonded OH groups), 2940–2900 cm^−1^ (aliphatic C–H stretching), 1720–1700 cm^−1^ (C–O stretching and COOH-OH deformation), and 1099 cm^−1^ (polysaccharide C–O stretching or silicate impurity Si–O stretching) were particularly noticeable in the pure FA, whereas in the FTIR spectra of the lyophilized NE (KBr), the bands were observed at 3398.72 (-OH), 3289.74 (-OH), 2905.89 (C-H, stretching), 2292 (O=C=O, stretching), 2058.13 (C-H, bending), 1738.90 (C=O, ester), 1648.24 (C=O, stretching), 1424.49 (C-H bending, methyl), 1287.54 (C-H, rocking), 1083.08 (-C-O), 928.76 (C-H wagging, alkene), and 874.76 cm^−1^ (C=C, bending). Collectively, a slight shift in the peaks was observed in the spectra of the lyophilized NE as compared to the pure drug and fulvic acid, thus indicating its amalgamated/complexed nature [41]. In the case of the mannitol (cryoprotectant), the peaks were very close to the NE spectra, as it was used in excess to ensure an effective lyophilization of the NE.

#### 2.5.5. X-ray Diffraction

**Figure 8:**
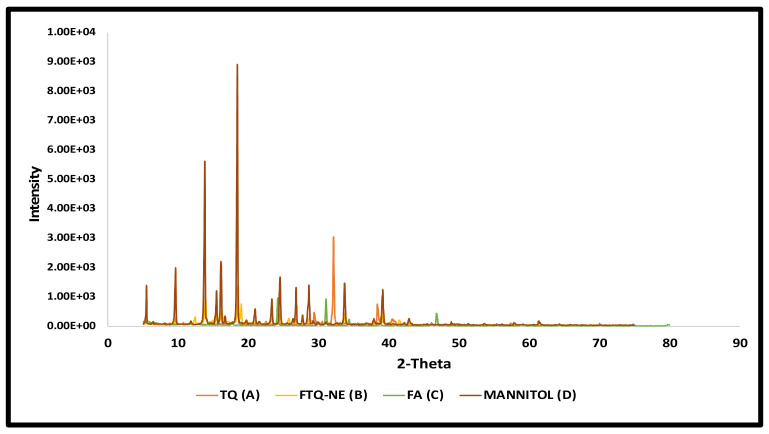
Overlay of X-ray diffractograms.

As shown in Figure 8, the XRD spectra of the pure drug, TQ, revealed a crystalline form with sharp peaks at the 2θ values 25° and 35°. The FA showed small amorphous peaks at 26°and 32°. The lyophilized formulation had no prominent peaks and had to be converted from a crystalline to an amorphous form. A few large peaks can also be seen at the 2θ values 15° and 18°. These peaks belong to the mannitol, which was also included as a cryoprotectant during the lyophilization process, but the peaks were reduced to an amorphous nature in the formulation. As a result, the lyophilized formulation might be described as amorphous, and this conclusion was supported by the DSC patterns, as discussed earlier in this section [42].

### 2.6. Surface Morphology

**Figure 9:**
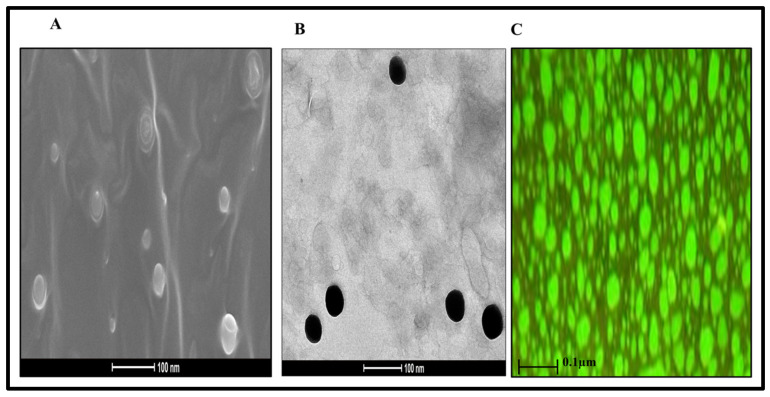
(**A**) SEM, (**B**) TEM, and (**C**) fluorescent microscopy of the optimized FTQ-NE.

The three different techniques were employed to analyze the structure and surface morphology of the final formulation. They collectively revealed a circular, globule-like structure with a size range below 100 nm (Figure 9). Furthermore, the obtained size range corroborated well with the observations of the zeta sizer. Thus, the optimized formulation is acceptable in terms of its shape and size, i.e., nanometric and globular size, respectively, for an efficient topical delivery permeation and retention [43].

### 2.7. Characterization of the FTQ-NEG

#### 2.7.1. Spreadability and Extrudability

Various placebo gel preparations with different concentrations of Carbopol 971^®^ (conc. range) were evaluated, and the concentration of 1% showed acceptable results. The optimized FTQ-NEG did not have a coarse texture and was smooth and homogenous. The pH of the optimized gel was 5.8 ± 0.32, which could be inferred as safe and non-irritating to the skin. The spreadability and extrudability mainly contribute to an easy application at the desired site and expulsion from the packaging, respectively. Thus, they are critical for a high patient compliance. The optimized FTQ-NEG has a spreadability of 15.38 ± 1.65 g.cm/s and extrudability of 5.68 ± 1.03 g [44].

#### 2.7.2. Texture Analysis of the Placebo and Formulation

**Figure 10:**
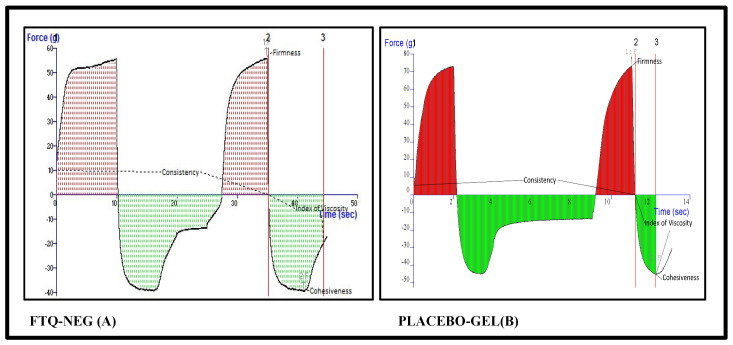
Texture analysis of the FTQ-NEG formulation (**A**) and placebo gel (**B**).

The textural characteristics were assessed for both the placebo and optimized formulation, and the corresponding results are presented in Figure 10. The optimized FTQ-NE had a cohesiveness of 39.91 gm, which determines its ability to adhere under the applied tensile stress. The firmness of the FTQ-NE was 56.25 gm, which mainly contributes to its structural integrity against a compressive force. The consistency variables at 444.88 g.s and index of viscosity at −302.39 g.s represent the fixed/definite/constant state and thickness, respectively. The aforementioned textural properties are crucial for the formulation’s stability, packaging, and ease of use [45].

### 2.8. Drug Release/Permeation Profile

#### 2.8.1. In Vitro Drug Release Study

**Figure 11:**
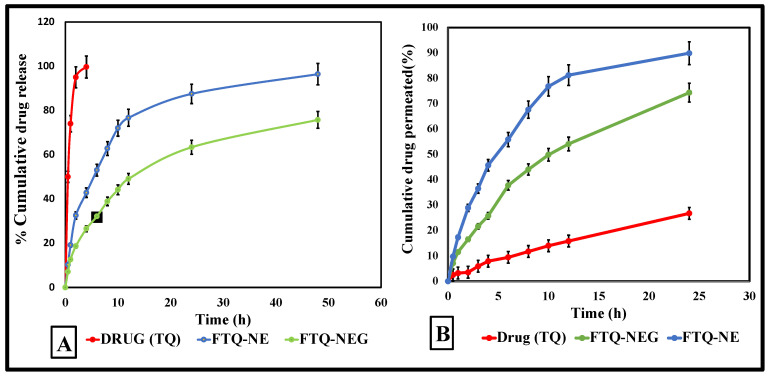
(**A**) In vitro drug release of TQ, FTQ-NE, and FTQ-NEG. (**B**) The ex vivo skin permeation release of the free drug TQ, FTQ-NEG, and FTQ-NE.

The cumulative release profiles for each optimized formulation, FTQ-NE and FTQ-NEG, and the pure drug TQ were performed using a dialysis membrane, calculated, and compared graphically, as presented in Figure 11A. The % cumulative drug release (%CDR) for the different formulation types was found to be 96.41 ± 4.15% (FTQ-NE), 75.76 ± 3.62% (FTQ-NEG), and 99.64 ± 3.22% (pure drug) after 48 h (Figure 11A). the optimized FTQ-NEG displayed a biphasic release behavior with a rapid release pattern in the initial 4 h, followed by a slow release afterwards. The initial fast release could be due to the presence of the drug on the formulation surface, and the following slow release may be due to drug entrapment in the gel matrix, which may hinder the diffusion. Additionally, the increased viscosity of FTQ-NE due to its incorporation into the gel matrix may impede the diffusion process. As mentioned in the literature, NE formulations have various rate-controlling drug release mechanisms, such as diffusion, swelling, and erosion [46]. To pinpoint the mechanism for the present formulation, different release kinetic models were employed, such as zero-order, first-order, and Higuchi models, and the drug release was diffusion-dependent (Higuchi model, R^2^-0.9704). The Higuchi models encourage the release of low-soluble APIs dispersed in a uniform semi-solid or solid matrix, which behaves as a diffusion medium.

#### 2.8.2. Ex Vivo Skin Permeation Study

The ex vivo permeation studies were performed using freshly excised mice skin (the hair was removed before sacrifice). The obtained permeation flux of the FTQ-NE (4.89 μg/cm^2^/h) was significantly higher (*p* < 0.0001) compared to FTQ-NEG (3.64 μg/cm^2^/h) and the pure drug (1.77 μg/cm^2^/h) (Figure 11B). The incorporation of FTQ-NE into a gel system as a carrier not only contributes to an easy delivery and permeation but also improves the formulation stability compared to non-gelling systems. In addition, the nanoscale size of a formulation complements the permeation through the innate layers of the skin, simultaneously resulting in an increased drug retention. In general, the present study evaluated the presence of the drug in the layers of skin for an extended or desired time, which can also determine its dosing frequency [47].

### 2.9. Confocal Laser Scanning Microscopy

**Figure 12:**
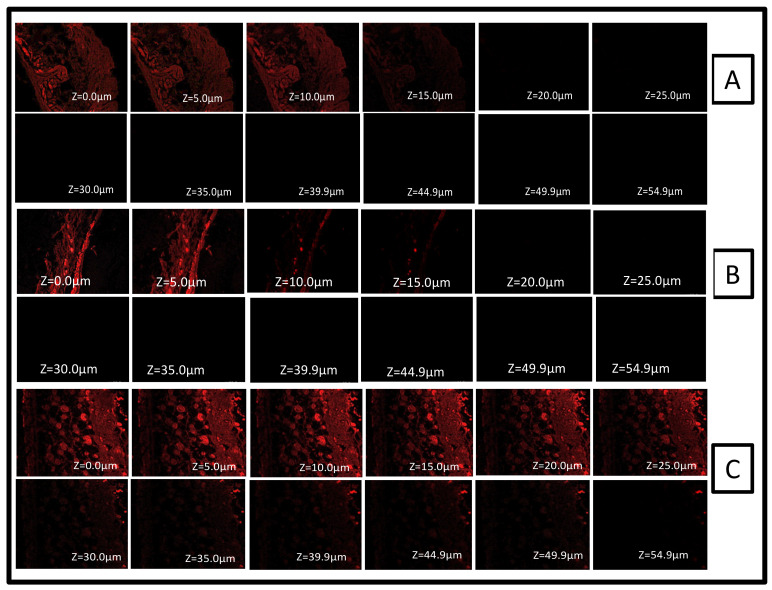
Confocal microscopic studies of mouse skin treated with (**A**) Rhodamine B hydroalcoholic solution, (**B**) drug solution with rhodamine dye, and (**C**) FTQ-NEG with rhodamine.

The confocal microscope imaging of the mice skin samples treated ex vivo revealed a localized drug/formulation penetration in the stratum corneum layer of the epidermis. The drug solution and formulation (FTQ-NEG) treated with a fluorescent dye (Rhodamine B) showed penetrations of 15 µm and 54.9 µm, respectively. In contrast, the hydroalcoholic solution of Rhodamine B showed a penetration of 12 µm (Figure 12A). Furthermore, the midway portion of the treated skin samples (ranging from 5–25 µm) indicated a high fluorescence intensity (Figure 12C), thus signifying a higher formulation retention. Notably, the thickness of the mice stratum corneum is 3–9 µm, and that of the epidermis is 9–29 µm. While the human skin stratum corneum thickness is 10–30 µm and the epidermis is 1500 µm thus, a similar effect on in vivo/clinical samples could be envisaged [48].

### 2.10. Stability Study

The ultrasonication method (code: FTQ-NE^+US^) was used to develop the FTQ-NE, which was reported to be stable for 15 weeks (Table 7). In the optimized FTQ-NE prepared without ultrasonication, phase separation occurred after only 7 weeks (code: FTQ-NE). The Z-average particle diameter and PDI of FTQ-NE^+US^ at the beginning (0 months) were 72.34 ± 2.43 nm and 0.135 ± 0.015, respectively. The diameter of the droplets expanded rapidly from 0 to 3 weeks, most likely due to their significant Ostwald ripening rate. In the first three weeks, the turbulence created accelerated the mobility of the droplets and tightened the connections between them, leading to molecular diffusion and Ostwald ripening. The PDI of the formulation likewise increased, showing a variation in the droplet size uniformity. According to the droplet size measurements, the droplet size increased a little after three and seven weeks, indicating a stable formulation. However, after 13 weeks, there was another increment in the droplet size but no evidence of phase transition. FTQ-NE had an initial particle size of 97.12 ± 4.21 nm and a PDI of 0.319 ± 0.072, respectively. The droplet size increased dramatically to 131.3 ± 7.02 nm after one week, and the PDI improved (0.123 ± 0.02). After seven weeks, the average droplet size rose above 200 nm, and phase transition was observed in FTQ-NE after seven weeks [49]. Micelle swelling occurs due to this process, and any micelles that are not absorbed remain in the formulation. Micelles disrupt the stability by causing droplet agglomeration. There was a significant difference between the Z-averages of FTQ-NE^+US^ and FTQ-NE in the stability investigation. This is because their Ostwald ripening processes varied. In conclusion, the better storage stability observed in FTQ-NE^+US^ is likely due to a lower Ostwald ripening rate [50]. The sluggish Ostwald ripening of FTQ-NE^+US^ could be attributed to the lack of micelles and the initially reduced droplet size. Therefore, the formulation remained stable for 15 weeks, despite its low zeta potential (−2.83 ± 0.15 mV). Tween-80, a non-ionic surfactant, also aids in the steric stabilization of the nanoemulsion and thus its stability [51]. According to the stability study, a nanoemulsion that is ultrasonic-assisted and QBD-validated enhances the nanoemulsion’s long-term stability.

## 3. Conclusions

The purported work explored a fulvic acid (peat sourced) based thymoquinone nanoemulsion gel/nanoemulgel using ultrasonic and “Quality by Design” (QbD) approaches. The combination of TQ with a functional excipient such as FA, incorporated into a nanoemulgel, is a potentially successful formulation approach, whereby the “pharmacological goodness” of TQ can be harnessed, along with that of FA, for the intended applications of the topically effective formulation. As envisaged, the excellent pharmaco-technical attributes of the FTQ NEG^+US^ nanoemulgel, with its pharmaceutically tailored properties, such as the enhanced solubility and consequent improved IVR, provided a better permeability through the mice skin model, and its locoregionally effective TQ bioavailability can support its clinical translation into an alternative therapy for challenging disease, such as psoriasis. Being fortified with the additional functionality of FA, the potential of the optimized formulation is expectably higher and worthy of investigation in preclinical and, eventually, clinical settings.

## 4. Materials and Methods

TQ and Rhodamine B were obtained from Sigma-Aldrich (Darmstadt,Germany). FA was acquired as a gift sample from New Zealand Fulvic limited. The ex-gratia samples of Transcutol P, Labrasol, Plural, and Capryol 90 were provided by Gattefosse India Pvt. Ltd. The other samples of polyethylene glycol, PEG 200, Tween 80, and Tween 20 were obtained from Sisco Research Laboratories Pvt. Ltd. (SRL, Mumbai, India). Carbopol 971^®^ was supplied by SD Fine Chemicals India Pvt. Ltd. (Mumbai, India). Merck Pvt. Ltd., Mumbai, provided HPLC-grade acetonitrile and methanol. The HPLC water and triethanolamine were provided by Loba Chemie Pvt. Ltd. (Mumbai, India). CDH provided lavender oil, kalonji oil, sunflower oil, castor oil, and olive oil (Delhi, India). All other chemicals, solvents, and reagents employed were of analytical quality.

### 4.1. HPLC Analytical Method

For the analytical studies, a previously reported method was used [52]. The description of the method is as follows: a ratio of mobile phase acetonitrile: water of 75:25 (*v*/*v*), a C-18 silica column (Waters, 5-micron, 250 × 4.0 mm, 5 μm particle size), flow rate of 1 mL/min, injection volume of 20 μL, and detection wavelength of 254 nm.

### 4.2. Screening of Excipients

The formulation development and management of the stability of an NE critically rely on the successful screening of various excipients, such as oil, surfactants, and co-surfactants. Therefore, multiple oils such as kalonji, castor, olive, lavender, sunflower oil, and Capryol 90 (liquid lipid) were screened for the present formulation based on the drug solubility and miscibility [53]. The surfactants and co-surfactants considered for screening were Labrasol, PEG-200, PG, Tween 80, Tween 20, and Transcutol P based on their respective HLB values and ionic characteristics. The procedure, followed with a few modifications, required the use of an excess amount of TQ (drug) added with 1 mL of the excipients (oil or surfactant) to a tube, which was then vortexed (Remi CM-101 cyclomixer; 72 h; 25 °C). Following centrifugation at 3000 ± 50 rpm for 10 min, an excess amount of the drug was allowed to settle, and the supernatant was obtained using a Remi R8C Laboratory centrifuge. The supernatant was then diluted with methanol following its filtering through a 0.22 µm syringe filter and vortexed. The diluted samples were then subjected to a UV spectrophotometer at λmax 254 nm, and the amount of solubilized drug was measured [54].

### 4.3. Formulation Development

#### 4.3.1. Preparation of a Placebo Formulation

The aqueous microtitration process was used to formulate the placebo NE and, subsequently, we evaluated the pre-selected oils with the surfactant: co-surfactant mixture (Smix), aided by a Remi cyclomixer (vortex). The selected oil and Smix were mixed and vortexed to form a clear blend, followed by dilution using double-distilled water [31]. The objective of this step was to check the compatibility/miscibility of the oils with the Smix and to optimize the amount of Smix using various ratios (1:1, 1:3, 1:4, 2:1, 3:1, 4:1), thus aiding the formation of a clear NE (Placebo) in terms of its visual clarity and turbidity after each dilution. Furthermore, the oil and Smix ratio was also varied, including 1:9, 1:8, 1:7, 1:6, 1:5, 1:3.5, 1:2, 2:8, 3:7, 4:6, 5:5, 6:4, 7:3, 8:2, and 9:1, to achieve a larger NE region and plot a pseudo-ternary phase diagram [55].

#### 4.3.2. Preparation of FTQ-NE

The abovementioned NE was prepared using a high-energy ultrasonication method. First, the required amount of TQ was dissolved in a pre-selected oil, followed by solubilization in a Smix. Next, the obtained blend was microtitrated with double-distilled water containing FA to produce a coarse pre-emulsion. This pre-emulsion was then subjected to an ultrasonic processor UP50H^®^ (Hielscher-Ultrasound Technology Teltow, Germany) at a frequency of 30 kHz and amplitude of 40% for a duration of 10 s to yield a nanosized formulation [56]. During the ultrasonication process, the formation of cavitation results in an increase in temperature, which is avoided by placing the sample container in an ice-filled beaker [57].

#### 4.3.3. Optimization of Prepared FTQ-NE: DoE

Design of Expert (DoE) software (version 132.0.4, Stat-Ease, Minneapolis, MN, USA) was employed to optimize the batches of FTQ-NE based on pre-selected independent and dependent variables. For the present formulation, the independent variables and dependent responses selected were the oil concentration (%), Smix ratio (%), sonication time (s) and particle size (nm), polydispersity index, and transmittance (%), respectively. In addition, the ternary phase diagrams were utilized to select the high, medium, and low levels of the oil and surfactant concentrations, as required in DoE. The optimization design followed was the Box–Behnken design (BBD), a response surface methodology approach used to predict the effects of independent variables on dependent responses [58,59]. A total of 17 randomized runs were acquired and compared via BBD to obtain an optimized formulation.

### 4.4. Thermal Stability Studies

Thermal stability studies of the prepared FTQ-NE were performed to analyze the phase separation, transparency, stability, and droplet size using the process described in [60], with a few modifications.

#### 4.4.1. Heating Cooling Cycle

The developed FTQ-NE formulations were subjected to six cycles at temperatures ranging from 4 to 45 °C to determine the formulation stability. It should be noted that storage for no less than 48 h at each temperature should be ensured.

#### 4.4.2. Centrifugation

The prepared FTQ-NE formulations were centrifuged at 4000 rpm for 30 min, and those with no phase separation were selected and further subjected to a freeze–thaw process.

#### 4.4.3. Freeze–Thaw Cycle

Following centrifugation, freeze–thaw processes at varying temperatures ranging from −21 to +25 °C were employed to preserve the FTQ-NE formulations, with no phase separation (temperature storage for no less than 48 h).

### 4.5. Characterization of the Optimized FTQ-NE

#### 4.5.1. Dilution Test

To classify the type of NE, water was added to a *w*/*o* nanoemulsion, and oil was added to an *o*/*w* nanoemulsion. Such an addition might cause either cracking or phase separation in an emulsion.

#### 4.5.2. Filter Paper Test

Another test used to classify the type of NE is the use of filter paper, as the *o*/*w* nanoemulsion spreads swiftly, whereas the *w*/*o* nanoemulsion migrates slowly.

#### 4.5.3. Cobalt Chloride Test

Filter paper soaked in CoCl_2_ solution and allowed to dry changes its color from blue to pink as the solution evaporates, resulting in an *o*/*w* emulsion.

#### 4.5.4. Transmittance

The %transmittance value refers to the transparency/clarity of the prepared FTQ-NE, with no visual signs of turbidity. The higher the transmittance is, the finer the formulation will be, with less scattering of the incident radiations at λmax 650 nm, as observed through a UV spectrophotometer (Shimadzu, UV-1601, Kyoto, Japan). No dilutions were employed for the above test, and the double-distilled water was kept blank [61].

#### 4.5.5. Refractive Index (RI) and Viscosity

The RI and viscosity of the optimized FTQ-NE with no prior dilutions were examined using an Abbe-type refractometer (Analytic Jena, GmbH Jena, Germany) and a Brook field viscometer (Brookfield Engineering LABS, Stoughton, MA, USA), respectively [62].

#### 4.5.6. Particle Size, Polydispersity Index (PDI), and Zeta Potential

The particle size of the optimized FTQ-NE was evaluated using a dynamic light scattering technique (DLS) (Malvern Zetasizer, Nano ZS Worcestershire, UK). The following parameters were maintained before and during the analysis: the formulation was diluted at 1:50, the system temperature was maintained at 25 °C, and the scattering angle was 90° [63].

#### 4.5.7. Differential Scanning Calorimetry (DSC)

The DSC analysis of the pure drug (TQ), FA, and lyophilized sample (using 5% *w*/*v* mannitol) of the optimized FTQ-NE formulation was performed using a Pyris 6 DSC, Perkin Elmer, USA. A brief procedure followed for each sample. A 2 mg sample was taken and subjected to a temperature range of 25–350 °C. The temperature was increased at a rate of 10 °C/min, and the nitrogen flow was maintained at 60 mL/min [64].

#### 4.5.8. Fourier Transform Infrared Spectroscopy (FTIR)

The FTIR analysis of the pure drug (TQ), FA, and lyophilized sample (using 5% *w*/*v* mannitol) of the optimized FTQ-NE formulation was performed using FTIR spectroscopy. First, a precisely weighed quantity of the aforementioned samples (5 mg) was combined with potassium bromide (KBr) in a ratio of (1:1) (different pellets were used for each sample) and then compressed using a hydraulic press to form a flat pellet. The obtained pellets were then scanned between an infrared spectrum of light with a wavenumber of 4500–500 cm^−1^ [65].

#### 4.5.9. X-ray Diffraction (XRD)

The X-ray diffraction of the pure TQ, FA, and lyophilized sample (using 5% *w*/*v* mannitol) of the optimized FTQ-NE formulation was performed using an X-ray diffractometer (Philips X-ray generator Poznan, Holland) at a 35 kV electric potential, 30 mA voltage, and detector angle of 2θ (10–70 degree) [66].

### 4.6. Surface Morphology Studies of an Optimized FTQ-NE

#### 4.6.1. Transmission Electron Microscopy (TEM)

The optimized FTQ-NE formulation was examined by TEM (Philips Briarcliff Manor, Briarcliff Manor USA). For the sample preparation, copper grids were inserted into a dust-free paraffin film with forceps, and a drop of the sample (pre-diluted, 1:100) was placed in it using a micropipette. In the same paraffin sheet, 2% of phosphotungstic acid (contrasting agent) was also placed and immersed in the copper grid. A couple of minutes later, the copper grids were removed, dried using a Whatman filter paper, and subjected to TEM analysis [67,68].

#### 4.6.2. Scanning Electron Microscopy (SEM)

A HITACHI Ion Sputter E-1010 (Hitachi, Tokyo, Japan) was used to conduct the SEM microscopy of the refined and optimized FTQ-NE formulation. Before imaging, the samples were platinum-coated, bonded to an aluminum glass plate using a double-sided carbon adhesive, and photographed with a VE-7800 scanning electron microscope [69].

#### 4.6.3. Fluorescent Microscopy

The optimized FTQ-NE formulation samples were previously diluted to 10-fold the initial concentration with water, stained with Rhodamine B, a water-soluble fluorescent dye, and analyzed using a fluorescence microscope [70].

### 4.7. Preparation of a Drug-Loaded Nanoemulgel Using an Optimized FTQ-NE

The optimized FTQ-NE formulation was prepared as an emulgel with varying proportions of the gelling ingredient, Carbopol 971 0.5% to 1% (*w*/*v*). The preparation steps followed a dispersion of Carbopol971^®^ in distilled water, with continuous stirring. The resultant formulation was sonicated for 15 min to eliminate any air bubbles, followed by the addition of sodium benzoate (a preservative) to obtain a homogenous nanoemulgel dispersion (NEG) [71].

### 4.8. Physicochemical Characterization of NEG

#### 4.8.1. Spreadability

A 0.5 g NEG sample was placed on a glass plate pre-marked with a circle of 1 cm in diameter, and another glass plate loaded with a 500 g weight was placed above it for 5 min. An increase in the diameter of a gel surface can indicates its spreadability using the following formula: W∗L/t = S

where:

S—spreadability (g/s)

W—weight on the glass plate (g)

L—length travelled by the glass plate

t—time taken (s)

The test was performed in triplicate, and a linear scale was used to determine the extension required to separate the glass plates [72].

#### 4.8.2. Extrudability

The measurement of extrudability determines the ease of expulsion of a formulation from a tube, i.e., 0.5 cm of the formulation in 10 s. The crimped end of a closed collapsible tube with a pre-weighed amount of the optimized NEG was taken, and a firm pressure was applied. After a certain length of time, the pressure was released, and the tube cap was removed to note the amount of extruded gel using a linear scale. The higher the extruded quantity is, the higher the extrudability will be [73].

#### 4.8.3. Texture Analyzer

A glass jar containing the NEG sample was placed on a flat testing surface to avoid premature triggers and air bubbles. The texture profile assessment of the firmness, cohesiveness, consistency, and viscosity indices was conducted in a compression mode using a texture analyzer (TA. Plus, Stable Micro Systems, Surrey, UK). The results were noted as force–time graphs to determine several mechanical characteristics of the NEG [74].

#### 4.8.4. Homogeneity and pH

Visual inspection was used to determine the homogeneity of the optimized NEG using a clear glass container. The pH was identified using a digital pH meter (HI 98107, Hanna Instruments Navi Mumba, India) coupled with a glass microelectrode at a temperature of 25 ± 1 °C and equilibration time of 1 min [75].

### 4.9. Drug Release/Permeation Profile

#### 4.9.1. In Vitro Drug Release Study

The release profile of the pure drug (TQ), FTQ-NE, and NEG formulations (containing a 5 mg equivalent of TQ) was performed through a pre-activated dialysis bag (m.wt. 12,000 Dalton). The test media used was phosphate saline buffer (pH 5.8) (250 mL) at 600 rpm and temperatures of 37 ± 0.5 °C. Then, 2 mL samples were collected at pre-determined time intervals and immediately replaced with an equal amount of dissolution media to balance the sink conditions. The aliquot collected was then filtered, diluted, and analyzed using a pre-validated HPLC method at wavelength of 254 nm [76].

#### 4.9.2. Ex vivo Skin Permeation Study

A hairless sample of mice skin was maintained in a Franz diffusion cell to assess the permeation pattern of the prepared formulations and pure drugs. A total of 10 mL of phosphate-buffered saline (pH 5.8) was added to the receptor compartment as a diffusion media, maintained at 37 ± 0.5 °C with a constant stirring speed of 600 rpm. At predefined time intervals, the aliquot (2 mL) was collected and replaced with a new volume of dissolving medium. The collected aliquot was further filtered, diluted, and analyzed using a pre-validated HPLC method at wavelength of 254 nm [77].

### 4.10. Confocal Laser Scanning Microscopy

The treated skin tissues were placed between the donor and receptor chambers of the Franz diffusion cell for the tissue uptake assessment. Rhodamine B aquatic solution (0.03 %*w*/*v*), TQ solution, and the formulation (FTQ-NEG) with Rhodamine were used in the donor compartments, whereas phosphate-buffered solution was used in the receiver compartments. After 24 h, the skin samples were obtained and washed thoroughly with distilled water and alcohol. The skin samples were then positioned on slides with the stratum corneum pointing upwards and identified with an argon laser beam at an excitation of 540 nm and emission of 625 nm, respectively, using a fluorescence microscope (Olympus FluoView TM FV1000, Hamburg, Germany) [78].

### 4.11. Storage Stability of the Optimized Nanoemulsion

The stability investigation of the optimized FTQ-NE was conducted at room temperature (25 °C ± 1 °C) for 15 weeks. Two drug formulations were evaluated for their stability to determine whether sonication affects the long-term storage. One consisted of an optimized NE without sonication, and the other was an ultrasonically customized NE. The size distribution was the parameter chosen for the stability studies. After completing 0, 1, 3, 7, 10, 13, and 15 months of storage, variations in the nanoemulsion droplet size were investigated [79].

## Figures and Tables

**Table 1:** Screening of Oils, Surfactants, and Co-Surfactants Based on Miscibility.

Scheme	Oils	Surfactants	Co-Surfactants	Inference
1	Lavender	Tween 80	Transcutol-P	No Phase separation
2	Sunflower	Tween 80	Transcutol-P	Phase separation
3	Kalonji	Tween 80	Transcutol-P	No phase separation
4	Castor	Tween 80	Transcutol-P	Phase separation
5	Olive	Tween 80	Transcutol-P	Phase separation
6	Sunflower	Labrasol	Transcutol-P	No phase separation
7	Kalonji	Labrasol	Transcutol-P	No phase separation
8	Lavender	Labrasol	Transcutol-P	No phase separation
9	Olive	Labrasol	Transcutol-P	No phase separation
10	Castor	Labrasol	Transcutol-P	No phase separation

**Table 2:** BBD-based FTQ nanoemulsion with independent and dependent variables.

Run	Oil (%)	Smix (%)	Sonication Time (s)	Particle Size (nm)	PDI	Transmittance (%)
1	2.5	12	20	99.76	0.23	94.65
2	1.75	12	10.5	72.34	0.12	99.01
3	1	16	10.5	32.26	0.24	99.42
4	1	12	20	43.12	0.23	94.24
5	1	8	10.5	36.26	0.22	99.51
6	1.75	12	10.5	72.34	0.12	98.19
7	1.75	8	20	60.52	0.26	98.41
8	1	12	1	49.22	0.21	96.94
9	1.75	16	20	56.61	0.29	98.58
10	1.75	12	10.5	72.34	0.12	98.98
11	1.75	12	10.5	72.34	0.12	98.99
12	2.5	16	10.5	92.63	0.23	99.19
13	2.5	12	1	108.53	0.24	94.9
14	1.75	16	1	65.43	0.28	99.62
15	1.75	12	10.5	72.34	0.16	98.79
16	1.75	8	1	66.42	0.26	98.51
17	2.5	8	10.5	93.68	0.25	97.42

**Table 3:** Selected independent variables and their levels.

Independent Variables	Levels		
	Low	Medium	High
X1 = Oil (%)	1	1.75	2.5
X2 = Smix (%)	8	12	16
X3 = Sonication time (s)	1	10.5	20

**Table 4:** Regression analysis summary for responses Y1 (particle size in nm), Y2 (PDI), and Y3 (%transmittance).

Quadratic Model	%CV	R^2^	Adjusted R^2^	Predicted R^2^	SD
Particle size (Y1)	0.36	0.9999	0.9999	0.9991	0.24
PDI (Y2)	7.19	0.9713	0.9344	0.8712	0.01
% Transmittance (Y3)	0.38	0.9802	0.9547	0.8253	0.37
Y1 = Particle size + 72.34 + 29.22 × A − 1.24 × B − 3.7 × C + 0.7375 × AB − 0.6675 × AC − 0.73 × BC + 2.14 × A^2^ − 10.77 × B^2^ + 0.6775 × C^2^					(1)
Y2 = PDI + 0.1280 + 0.0063 × A + 0.0063 × B + 0.0025 × C − 0.0100 × AB 0.0075 × AC + 0.0025 × BC + 0.031 × A^2^ + 0.0760 × B^2^ + 0.0685 × C^2^					(2)
Y3 = Transmittance + 98.79 − 0.4937 × A + 0.37 × B − 0.5113 × C + 0.465 × AB + 0.6125 × AC − 0.235 × BC − 1.75 × A^2^ + 1.85 × B^2^ − 1.86 × C^2^					(3)

**Table 5:** Thermodynamic stability testing of the selected nanoemulsion formulations using heating–cooling (HC), freeze–thaw (FT), and centrifugation (Cent).

Smix Ratios	Formulation Coding	Percentage of Component % (*v*/*v*)			Observation			Inference
		Oil	Smix	Water	Heating–Cooling	Freeze–Thaw	Centrifugation	
Formulation P Smix = ratio 2:1	P-1 P-2 P-3 P-4	2.22 2 2.5 7.84	17.78 14 17.5 47.06	80 85 80 45.1	✓ ✓ × ✓	✓ ✓ × ×	✓ ✓ × ×	Passed Passed Failed Failed
Formulation Q Smix = ratio 3:1	Q-1 Q-2 Q-3 Q-4	1.67 1.87 2 2.86	13.33 13.12 14 17.14	85 85 85 80	× × ✓ ✓	✓ × ✓ ×	✓ × ✓ ×	Failed Failed Passed Failed
Formulation R Smix = ratio 4:1	R-1 R-2 R-3 R-4	2.78 3.13 2.5 2	22.22 21.88 12.5 18	75 75 85 80	✓ ✓ ✓ ×	✓ ✓ × ×	✓ ✓ × ×	Passed Passed Failed Failed

**Table 6:** Physical characterization studies of various nanoemulsion formulations.

Formulation Coding	Particle Size (nm) (Mean ± SD) (*N* = 3)		PDI (Mean ± SD) (*N* = 3)		pH (Mean ± SD) (*N* = 3)		RI (Mean ± SD) (*N* = 3)		Viscosity (mp) (Mean ± SD) (*N* = 3)	
	Blank	FTQ-NE	Blank	FTQ-NE	Blank	FTQ-NE	Blank	FTQ-NE	Blank	FTQ-NE
P-1	150.6 ± 1.34	224.7 ± 1.30	0.24 ± 0.05	0.28 ± 0.03	5.9 ± 0.34	6.1 ± 0.44	1.401 ± 0.006	1.402 ± 0.008	43.31 ± 3.24	43.78 ± 2.42
P-2	41.13 ± 1.45	72.34 ± 1.43	0.16 ± 0.02	0.13 ± 0.03	5.8 ± 0.25	5.8 ± 0.32	1.405 ± 0.003	1.405 ± 0.007	134.0 ± 6.42	138.5 ± 3.08
Q-3	100.13 ± 1.74	187.6 ± 1.23	0.18 ± 0.04	0.23 ± 0.02	5.6 ± 0.13	5.7 ± 0.25	1.402 ± 0.002	1.368 ± 0.004	104.0 ± 3.40	106.0 ± 3.49
R-1	106.3 ± 2.33	159.3 ± 1.57	0.38 ± 0.03	0.33 ± 0.04	6.4 ± 0.65	6.6 ± 0.27	1.405 ± 0.005	1.402 ± 0.004	52.60 ± 2.70	53.51 ± 2.02
R-2	112.14 ± 2.74	171.2 ± 2.13	0.15 ± 0.06	0.18 ± 0.02	6.3 ± 0.43	6.7 ± 0.14	1.403 ± 0.006	1.407 ± 0.004	157.2 ± 6.72	159.6 ± 5.52

**Table 7:** Stability study of the optimized FTQ-NE.

Duration (Week)	FTQ-NE (Before Sonication)		FTQ-NE^+US^ (After Sonication)	
	Particle Size (nm) (*n* = 3 ± SD)	PDI (*n* = 3)	Particle Size (nm) (*n* = 3 ± SD)	PDI (*n* = 3 ± SD)
0	97.12 ± 4.21	0.319 ± 0.072	72.34 ± 1.42	0.135 ± 0.014
1	131.3 ± 7.02	0.123 ± 0.02	95.03 ± 3.02	0.202 ± 0.035
3	145.2 ± 13.71	0.214 ± 0.032	105.8 ± 5.12	0.142 ± 0.046
7	241.4 ± 19.82	0.321 ± 0.057	108.2 ± 6.03	0.227 ± 0.013
10	Phase separation	Phase separation	112.3 ± 7.13	0.237 ± 0.016
13	Phase separation	Phase separation	125.3 ± 9.12	0.238 ± 0.024
15	Phase separation	Phase separation	135.8 ± 7.34	0.289 ± 0.023

## Data Availability

Not applicable.
